# Supplementary material for: Identifying feasible operating regimes for early T-cell recognition: The speed, energy, accuracy trade-off in kinetic proofreading and adaptive sorting
Source: PLoS One. 2018 Aug 16;13(8):e0202331. doi: 10.1371/journal.pone.0202331 (PMC6095552; doi:10.1371/journal.pone.0202331)
Supplement: S1 File — Definition of Model and parameter choices (A), Accuracy, Energy Consumption, Role of γ and Speed; Simulation Details for Phase Diagram (B). (PDF) [file pone.0202331.s001.pdf]

S1 file: Modeling and simulation

Wenping Cui and Pankaj Mehta

A. DEFINITION OF MODEL AND PARAMETER CHOICES

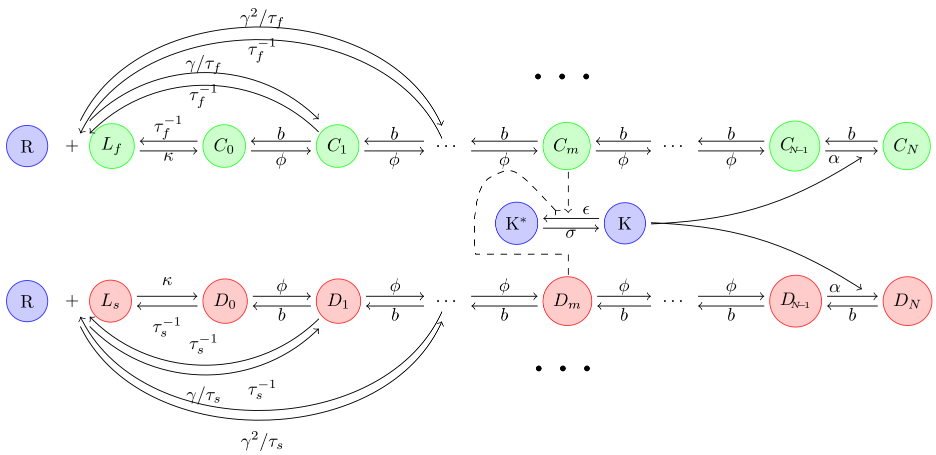

FIG. 1. Schematic overview of a general kinetic proofreading network.

A schematic of the model we are considering is shown in Fig. 1. As described in the main text, we denote a receptor-ligand complex that has been phosphorlyated n times by X_n with $X = C$ for foreign ligands and $X = D$ for self ligands. Furthermore, we denote the maximum number of phosphorylations as N. With this notation, using the law of mass action, we have

$$\begin{aligned}
\dot{C_0} &= \kappa R L_f - \left(\tau_f^{-1} + \phi\right) C_0 + b C_1 \\
\dot{C_n} &= \gamma^n R L_f/\tau_f + \phi C_{n-1} - (\phi + \tau_f^{-1} + b) C_n + b C_{n+1} \\
\dot{C_N} &= \gamma^N R L_f/\tau_f + \alpha K C_{N-1} - (b + \tau_f^{-1}) C_N \\
\dot{K} &= -\epsilon K (C_m + D_m) + \sigma (K_T - K) \\
\dot{D_0} &= \kappa R L_i - \left(\tau_s^{-1} + \phi\right) D_0 + b D_1 \\
\dot{D_n} &= \gamma^n R L_i/\tau_s + \phi D_{n-1} - (\phi + \tau_s^{-1} + b) D_n + b D_{n+1} \\
\dot{D_N} &= \gamma^N R L_s/\tau_s + \alpha K D_{N-1} - (b + \tau_s^{-1}) D_N
\end{aligned} \tag{1}$$

where $N > n > 0$, $R = R_T - \sum_{j=0}^{N}(C_j + D_j) \sim R_T$, $L_s = L_s^T - \sum_{j=0}^{N} D_j$ and $L_f = L_f^T - \sum_{j=0}^{N} C_j$. Typically, we set: $R_T = 10^4$, $L_f^T = L_s^T = 10^4$, $\kappa = 300s^{-1}$, $\sigma = 1s^{-1}$, $\epsilon = 2s^{-1}$, $K_T = 10^4$, $\alpha = 3 \times 10^{-4}$, $\gamma = 10^{-3}$, $\tau_s = 1s$ and $\tau_f = 10s$. Any deviations from this choice of parameter is explicitly noted.

Accuracy

At steady state, the error rate can be written as

$$\eta = \frac{D_N}{C_N} \tag{2}$$

In the presence of the kinase feedback $K = \frac{\sigma K_T}{\sigma + \epsilon(C_2 + D_2)}$, the set of eqs. (1) are no longer linear and but the steady-state solution can still be found easily using an iterative method.

Energy Consumption

The power dissipation is calculated based on the net flux and the chemical potential difference[1, 2]. We define the net flux $J_{\alpha,n}$, $i \in [s, f]$ at $X_n \rightleftharpoons X_{n+1}$ in the main pathway.

$$J_{i,n} = \begin{cases} \phi X_n - bX_{n+1}, \text{ for } 0 \leq n < N-1 \\ \alpha K X_{N-1} - bX_N, \text{ for } n = N \end{cases}$$

Considering the flux conservation, the power dissipation P_i can be written as

$$\begin{aligned} P_i &= k_B T J_{i,0} \ln \frac{\kappa R L_i^{free}}{\tau_i^{-1} X_0} + k_B T \sum_{n=0}^{N-2} J_{i,n} \ln \frac{\phi X_i}{b X_{i+1}} + k_B T J_{i,N-1} \ln \frac{\alpha K X_{N-1}}{b X_N} \\ &+ k_B T \sum_{n=0}^{N-2} (J_{i,n} - J_{i,n+1}) \ln \frac{X_{n+1}}{\gamma^{n+1} R L_i^{free}} + k_B T J_{i,N-1} \ln \frac{X_N}{\gamma^N R L_i^{free}} \\ &= k_B J_{i,0} \ln \frac{\kappa}{\tau_i^{-1}} + k_B T \sum_{n=0}^{N-2} J_{i,n} \ln \frac{\phi}{b\gamma} + k_B T J_{i,N-1} \ln \frac{\alpha K}{b\gamma} \end{aligned}$$

The total power dissipation is from the contribution of both foreign and self ligands: $P = P_s + P_f$.

Role of γ

In KPR, the reversible decay rate is ignored as it has extremely small value. Let $\gamma_{n,i}$ denote the rate at which a self ($i = s$) or foreign ligand ($i = f$) can directly form a complex at $n - th$ step of the KPR cascade (see Fig. 2 of main text). In such a reaction, the first $n - 1$ steps of the KPR cascade are bypassed resulting in lower accuracy. There are several natural choices for how to choose $\gamma_{n,i}$. One common choice in the literature is to assume that $\gamma_{n,i}$ is independent of n and given by $\gamma_{n,i} = \gamma/\tau_i$. However, with this choice never saturates the KPR accuracy bound for an N-step cascade, $\eta_{min} = \tau_s^N/\tau_f^N$, especially when N is large (see Fig 2).

For this reason, in this work we choose a step-dependent rate, $\gamma_{n,i} = \gamma^n/\tau_i$ ($i = s, f$), for directly forming a complex C_n and D_n This functional form is a direct consequence of assuming that there is a constant free energy difference $k_B T \log \phi/\gamma b$ per phosphorylation. Having a large γ will result in a bypassing of the proofreading steps and a high error threshold for any KPR-based circuit.

One choice o γ^n/τ_i. There are two reasons for this form: 1. the production rate from ligands and receptors to X_{n+1} should be smaller then the one to X_n as one more phosphorylation step is involved. If not, it is hard for the KPR circuit to achieve the theoretical limit, τ_s^N/τ_s^N. 2. it is also natural to assume the energy consumption is the same for each phosphorylation step.

The free energy difference between nth and n+1th phsphorlyation round can be calculated as:

$$\begin{aligned} \Delta G_n &= k_B T \log \left[\frac{\kappa}{\tau_i^{-1}} \frac{\phi^{n+1}}{b^{n+1}} \frac{\tau_i^{-1}}{\gamma^{n+1}/\tau_i} \right] - k_B T \log \left[\frac{\kappa}{\tau_i^{-1}} \frac{\phi^n}{b^n} \frac{\tau_i^{-1}}{\gamma^n/\tau_i} \right] \\ &= k_B T \log \frac{\phi}{\gamma b} \end{aligned} \tag{3}$$

Speed

The speed is defined by the mean first passage time(MFPT) for the foreign ligand. Here we mainly follow the procedures in Ref. [3]. The concentration vector is defined as $\mathbf{c} = [L_f, C_0, C_1, \ldots, C_N, p_f]$. An final 'dark' state is added because the response is only activated at the end and it can be treated as absorbing markov chain. Added this absorb state, it becomes an irreversible process, which is impossible to calculate the energy consumption. The transfer probability from C_N to the 'dark' state is W(irreversible). We set $W = 100s^{-1}$, a large value, which means the final step has little effect on MFPT. Without loss of generality, we begin with $N = 4$ and $m = 2$, which can be

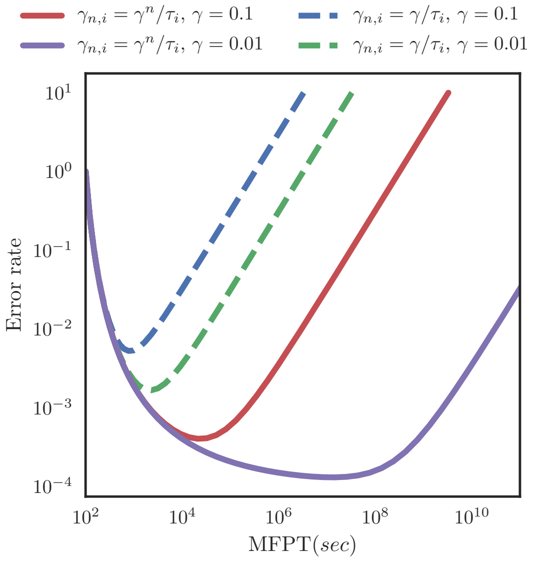

FIG. 2. Comparison between two different ways of parameterizing the rate $\gamma_{n,i}$: $\gamma_{n,i} = \gamma^n/\tau_i$ (solid lines) and $\gamma_{n,i} = \gamma/\tau_i$ (dashed lines). If we assume all reversible decay rates are the same, it is impossible to saturate the theoretical "Hopfield bound" for accuracy, $\tau_s^N/\tau_s^N = 10^{-4}$, when $\gamma_{n,i}$ is chosen according to the later scheme.

generalized other cases easily. The master equations *i.e.* eqs. (1) can be rewritten as $\dot{\mathbf{c}} = \mathbf{A}\mathbf{c}$ and

$$\mathbf{A} = \begin{bmatrix} -\kappa R - \sum_{j=1}^{4}\gamma^j/\tau_f & \frac{1}{\tau_f} & \frac{1}{\tau_f} & \frac{1}{\tau_f} & \frac{1}{\tau_f} & \frac{1}{\tau_f} & 0 \\ \kappa R & -\frac{1}{\tau_f} - \phi & b & 0 & 0 & 0 & 0 \\ \gamma/\tau_f & \phi & -\phi - \frac{1}{\tau_f} - b & b & 0 & 0 & 0 \\ \gamma^2/\tau_f & 0 & \phi & -\phi - \frac{1}{\tau_f} - b & b & 0 & 0 \\ \gamma^3/\tau_f & 0 & 0 & \phi & -\alpha K - \frac{1}{\tau_f} - b & b & 0 \\ \gamma^4/\tau_f & 0 & 0 & 0 & \alpha K & -b - \frac{1}{\tau_f} - W & 0 \\ 0 & 0 & 0 & 0 & 0 & W & 0 \end{bmatrix} \tag{4}$$

But eqs. (1) are not linear. The first order perturbation approximation is adapted and we can linearize (with bar denoting average) to get $\mathbf{c} = \bar{\mathbf{c}} + \delta\mathbf{c}$.

$$\delta\dot{\mathbf{c}} = \mathbf{A}'\delta\mathbf{c}, \quad \delta\mathbf{c} = [\delta L_f, \delta C_0, \delta C_1, \ldots, \delta C_N, p_f] \tag{5}$$

where $\mathbf{A}'$ is

$$\mathbf{A}' = \begin{bmatrix} -\kappa R - \sum_{j=1}^{4}\gamma^j/\tau_f & \frac{1}{\tau_f} & \frac{1}{\tau_f} & \frac{1}{\tau_f} & \frac{1}{\tau_f} & \frac{1}{\tau_f} & 0 \\ \kappa R & -\frac{1}{\tau_f} - \phi & b & 0 & 0 & 0 & 0 \\ \gamma/\tau_f & \phi & -\phi - \frac{1}{\tau_f} - b & b & 0 & 0 & 0 \\ \gamma^2/\tau_f & 0 & \phi & -\phi - \frac{1}{\tau_f} - b & b & 0 & 0 \\ \gamma^3/\tau_f & 0 & 0 & \phi + \frac{\alpha K \sigma C_3}{\epsilon + \sigma(C_2 + D_2)} & -\alpha K - \frac{1}{\tau_f} - b & b & 0 \\ \gamma^4/\tau_f & 0 & 0 & -\frac{\alpha K \sigma C_3}{\epsilon + \sigma(C_2 + D_2)} & \alpha K & -b - \frac{1}{\tau_f} - W & 0 \\ 0 & 0 & 0 & 0 & 0 & W & 0 \end{bmatrix} \tag{6}$$

Applying the Laplace transform, $\delta\mathbf{C}(s) = \int_0^\infty \delta\mathbf{c} e^{-st} dt$, the master equations can be rewritten as:

$$(s - \mathbf{A})\delta\mathbf{C}(s) = \delta\mathbf{c}(t = 0) = [1, \ldots 0]^T \tag{7}$$

The MFPT can be written:

$$T = \int_0^\infty t p_f(t) dt = -\frac{d}{ds}\int_0^\infty p_f(t) e^{-st} dt|_{s=0} = -W\frac{d\delta C_N(s)}{ds}|_{s=0} \tag{8}$$

which can be calculated numerically. It should be notified that the concentration and probability have the same master equations but a different pre-factor. When choosing the initial condition $[1, \ldots 0]^T$, the pre-factor is set to be 1 and $\delta C_N(s)$ solved from eq. (7) is exactly a probability distribution .

B. SIMULATION DETAILS FOR PHASE DIAGRAM

In this figure, we run 10^6 samples with random sets log uniformly chosen between $\gamma \in [10^{-4}, 10]$, $\phi \in [10^{-10}, 10^{10}]s^{-1}$, $b \in [10^{-15}, 10^{15}]s^{-1}$.

It can be observed that a large amount of red points distributes over regimes C and D with $\eta \sim 100$. This is because of $\gamma \sim 10$ and the inverse flux at the final step dominates. In the extreme case: b/ϕ is very large, $C_0 \simeq D_0$ will occupy most of products and free ligands L_s, L_f have little concentration.

$$\frac{L_s}{L_f} = \frac{D_0 \tau_s^{-1}}{C_0 \tau_f^{-1}} = \frac{\tau_s^{-1}}{\tau_f^{-1}}$$

As γ^N/τ_i dominates,

$$\eta = \frac{D_N}{C_N} = \frac{L_s}{L_f} \frac{\gamma^N/\tau_s}{\gamma^N/\tau_f} = \frac{\tau_f^2}{\tau_s^2} = 100$$

[1] T. L. Hill, *Free energy transduction and biochemical cycle kinetics* (Springer Science & Business Media, 2012).
[2] H. Qian, Annu. Rev. Phys. Chem. **58**, 113 (2007).
[3] G. Bel, B. Munsky, and I. Nemenman, Physical biology **7**, 016003 (2009).
