# Supplementary figures and images for: Identifying feasible operating regimes for early T-cell recognition: The speed, energy, accuracy trade-off in kinetic proofreading and adaptive sorting
Source: PLoS One. 2018 Aug 16;13(8):e0202331. doi: 10.1371/journal.pone.0202331 (PMC6095552; doi:10.1371/journal.pone.0202331)

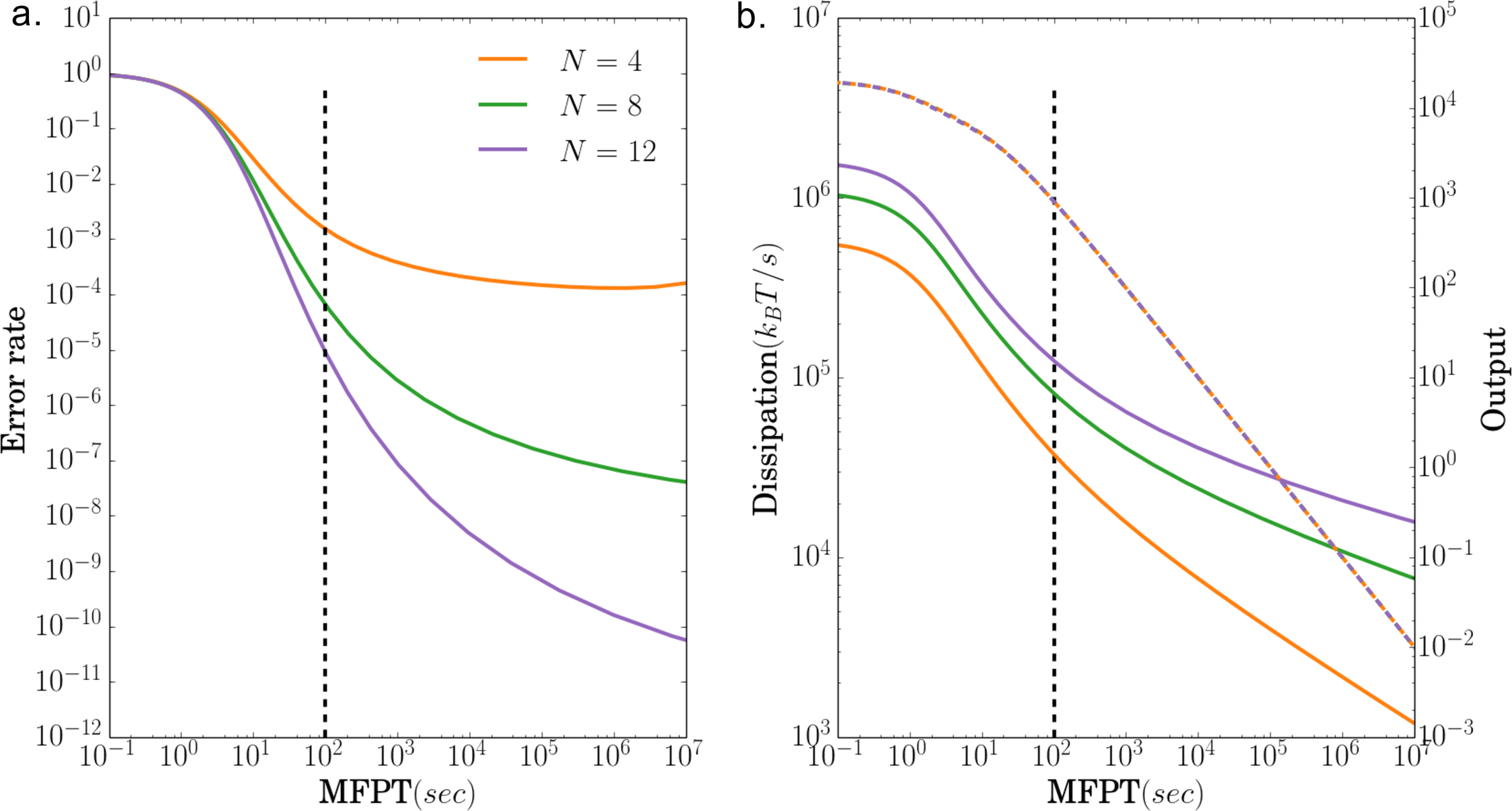

Supplement: S1 Fig — Here, we show simulations for the KPR-cascade when we vary the maximum number of phosphorylation steps N. Effects of the step size N in KPR: τs = 1s, τf = 10s, we change ϕ but keep b/ϕ = 0.01, γ = 10−3 fixed. The lines are for N = 4, 8 and 12. The vertical black dashed line is for time = 100s. (a): relation between accuracy and speed; (b): relation between dissipation(solid)/output(dashed) and speed. (TIF) [file pone.0202331.s002.tif]
